# Mental health and quality of life in patients with craniofacial movement disorders: A cross-sectional study

**DOI:** 10.3389/fneur.2022.938632

**Published:** 2022-09-21

**Authors:** Ming Yi, Jing Li, Gang Liu, Zilin Ou, Yanmei Liu, Jing Li, Yicong Chen, Yaomin Guo, Ying Wang, Weixi Zhang, Jinsheng Zeng, Chao Dang

**Affiliations:** ^1^Department of Neurology, The First Affiliated Hospital, Sun Yat-sen University; Guangdong Provincial Key Laboratory of Diagnosis and Treatment of Major Neurological Diseases; National Key Clinical Department and Key Discipline of Neurology, Guangzhou, China; ^2^Department of Ophthalmology, The Maternal and Child Health Care Hospital of Guangdong Province, Guangzhou, China

**Keywords:** craniofacial movement disorders, blepharospasm, hemifacial spasm, Meige syndrome, mental health, quality of life

## Abstract

**Background:**

Facial appearance and expressions influence social interaction. Hemifacial spasm (HFS), blepharospasm (BPS), and blepharospasm-oromandibular dystonia (BOD) are common forms of craniofacial movement disorders. Few studies have focused on the mental burden and quality of life (QoL) in patients with craniofacial movement disorders. Therefore, this study investigated mental health and QoL in these patients.

**Methods:**

This cross-sectional study included 90 patients with craniofacial movement disorders (HFS, BPS, and BOD; 30 patients per group) and 30 healthy individuals without craniofacial movement disorders (control group) recruited from October 2019 to November 2020. All participants underwent QoL and mental health evaluations for depression, anxiety, and stigma using the 36-item Short Form Health Survey (SF-36), Hamilton Anxiety Rating Scale (HAMA), Hamilton Rating Scale for Depression-24 (HAMD-24) and a questionnaire related to stigma.

**Results:**

Depression was diagnosed in 37 (41.11%) patients, whereas 30 patients (33.33%) had anxiety. HAMA scores were significantly higher in the BPS and BOD groups than in the control group. Nineteen patients (21.11%) experienced stigma and SF-36 scores were lower in various dimensions in the movement disorders groups compared to healthy controls. The role-physical and social function scores were significantly lower in the movement disorders groups than in the control group all *p* < 0.05. The vitality scores of the BPS group and mental health scores of the BPS and BOD groups were significantly lower than those of the control group. Correlation analysis showed that the eight dimensions of SF-36 correlated with education level, disease duration, HAMD score, and HAMA score (all *p* < 0.05). Regression analysis demonstrated that the HAMD score correlated with general health, vitality, social function, role-emotional, and mental health (all *p* < 0.05). The HAMA score correlated with body pain after adjusting for education level and disease duration.

**Conclusion:**

This study highlights the significant frequency of mental symptoms, including depression, anxiety, and stigma, which lower QoL in patients with craniofacial movement disorders.

## Introduction

Movement disorders in the facial region can be classified in various ways. Hemifacial spasm (HFS) and Meige syndrome are two common types of craniofacial movement disorders characterized by intermittent twitching of facial muscles (1, 2), which may lead to inconvenience and are associated with a mental burden. Although motor symptoms may be alleviated by clinical treatment, patients with craniofacial movement disorders experience psychological stressors, such as depression and stigma (3, 4). Furthermore, the disease-related quality of life (QoL), which affects subsequent management, is generally underestimated (5).

HFS is one of the most common craniofacial movement disorders (6), which is usually unilateral and originates from the periorbital musculature, then progresses to the perioral and other facial expression muscles (7). Meige syndrome is an another common type of craniofacial movement disorders characterized by blepharospasm (BPS) and oromandibular dystonia (8). Idiopathic blepharospasm and oromandibular dystonia may occur separately or together. Blepharospasm-oromandibular dystonia (BOD), which was first described by Meige in 1910 (9), is considered a type of Meige's syndrome (10, 11). The accepted pathophysiology of HFS is ectopic firing and ephaptic transmissions originating in the root exit zone of the facial nerve (12), while basal ganglia dysfunction is common in Meige syndrome (8). Although the pathophysiology of these diseases is different, the clinical symptoms and treatment experiences of these patients share multiple similarities. Microvascular decompression is a surgical intervention that provides relief by reducing compression of the facial nerve root in HFS (13). Deep brain stimulation of the globus pallidus interna has emerged as an alternative treatment option in Meige's syndrome patients (14). For non-surgical treatment, botulinum toxin has become an effective treatment for most patients (8, 15) and is supported by high-quality evidence for the treatment of craniofacial movement disorders (16). The prevalence of craniofacial movement disorders varies across different countries, with a large number of misdiagnoses and missed diagnoses, and is more common in women than in men (17). Age is an independent risk factor for craniofacial movement disorders, and the average time of disease onset is the fifth and sixth decades (1, 18). Spontaneous remissions are rare, occurring in <10% of patients (19). The etiology and pathogenesis of craniofacial movement disorders are not well understood; mental status, drug use, environmental triggers, and genetic predisposition may cause neuromodulation imbalances in the brain (14).

Psychiatric comorbidities are often neglected when physical symptoms can be effectively managed. Accumulating evidence suggest that depression and anxiety disorders appear to be related to the underlying disease processes of several dystonia syndromes (20, 21). Some studies report that psychiatric illness in patients with dystonia has a significant impact on patient QoL (22, 23). There are no published treatment trials for psychiatric illness in the context of movement disorders (24). Few studies have focused on the mental health and QoL of Chinese patients with HFS, BPS, and BOD. We hypothesized that mental health and stigma, which affect QoL and treatment outcomes, are often underestimated in patients with HFS, BPS, and BOD.

## Materials and methods

### Participants

We consecutively enrolled 90 patients with craniofacial movement disorders, including 30 with HFS, 30 with BPS, and 30 with BOD. All patients were identified from the outpatient dystonia clinic of our hospital from October 2019 to November 2020. The inclusion criteria were (1) age 18–80 years and (2) diagnosis of HFS or Meige syndrome according to the Guidelines for the Diagnosis and Treatment of Dystonia (25). Patients with Meige syndrome were classified as having either BPS or BOD. The exclusion criteria were (1) brain computed tomography or magnetic resonance images suggesting brain damage, (2) no other associated neurological diseases (e.g., epilepsy and myasthenia gravis), (3) secondary movement disorders, or (4) mental disorders diagnosed according to the Diagnostic and Statistic Manual of Mental Disorders, fifth edition.

Approval was obtained from the institutional ethics committee (the Research Ethics Committee of the First Affiliated Hospital of Sun Yat-sen University), and all participants provided their written informed consent before study participation.

### Assessment

All enrollees underwent the mental health assessment for anxiety, depression, stigma, and QoL.

Disease-related QoL was assessed using the 36-item Short Form Health Survey (SF-36), which comprises 36 questions covering eight dimensions related to physical functions and well-being (26). Physical functioning items included physical function (PF), role-physical (RP), role-emotional (RE), and social function (SF). Well-being included general health (GH), vitality (VT), body pain (BP), and mental health (MH).

Anxiety severity was assessed using the Hamilton Anxiety Rating Scale (HAMA), which consists of 14 items. Each item is scored on a scale of 0 (not present) to 4 (severe), with a total score range of 0–56, where <17 indicates mild severity, 18–24 indicates mild to moderate severity, and 25–30 indicates moderate to extreme severity (27). Depressive symptoms were assessed using the Hamilton Depression Rating Scale 24-Item (HAMD-24), which is a clinician-rated scale that assesses depressive symptoms. A HAMD-24 score of <8 points was defined as non-depression, whereas a score of ≥8 points was defined as depression. HAMD-24 scores of 8–19, 20–34, and ≥35 points were defined as mild, moderate, and severe depression, respectively (28). HAMD and HAMA have good reliability and validity (29). The questionnaire administered to evaluate stigma was a self-completed stigma scale developed in patients with epilepsy (30), which has a moderate to high internal consistency (Cronbach's α = 0.75). The scale assessed internal stigma regarding patient-perceived stigmatization and sense of shame and enacted stigma, which was measured by the patient's experience with discrimination and prejudice from the public, such as unemployment due to craniofacial movement disorders (30).

### Statistical analysis

Statistical analysis was performed using SPSS version 25 (IBM Corp., Armonk, NY). Descriptive data are presented as means ± standard deviations (SD), and categorical variables are presented as frequencies and percentages. All variables were compared using *t*-tests or Z-tests for continuous variables and the chi-square test or Fisher's exact test for categorical variables. Linear regression models were used to analyze associations among variables. *P*-values < 0.05 (two-sided) were considered statistically significant.

## Results

### Patient demographics

In total, 120 participants completed the questionnaires. Table 1 presents the patients' demographic characteristics. Of the 90 patients with craniofacial movement disorders, 78.89% were women and 21.11% were men. The average disease duration was 3.65 years, and 85.56% of patients had been treated with botulinum toxin.

**Table 1:** Participant characteristics.

**Variables**	**BPS (*n =* 30)**	**HFS (*n =* 30)**	**BOD (*n =* 30)**	**Control (*n =* 30)**	***P*-value**
	***n* (%)**	***n* (%)**	***n* (%)**	***n* (%)**	
Sex (male)	8 (26.67)	8 (26.67)	3 (10.00)	12 (40.00)	0.071
Age, years					0.0578
31–40	4 (13.33)	1 (3.33)	1 (3.33)	2 (6.67)	
41–50	5 (16.67)	6 (20.00)	5 (16.67)	5 (16.67)	
51–60	10 (33.33)	14 (46.67)	10 (33.33)	10 (33.33)	
>60	11 (36.67)	9 (30.00)	14 (46.67)	13 (43.33)	
Education					0.001
Less than high school	9 (30.00)	15 (50.00)	20 (66.67)	8 (26.67)	
High school	10 (33.33)	9 (30.00)	7 (23.33)	10 (33.33)	
College or higher	11 (36.67)	6 (20.00)	3 (10.00)	12 (40.00)	
HAMA	6.77(5.61)	4.93(3.60)	5.90(3.99)	3.13(2.96)	0.009
HAMD	8.07(5.73)	5.40(3.58)	7.47(4.42)	5.37(3.85)	0.106
Disease duration, years	3.45(3.29)	4.42(4.97)	3.07(3.02)		0.768
BoNT, years	6.55(4.58)	9.87(5.89)	6.18(4.91)		0.012

BPS, blepharospasm; HFS, hemifacial spasm; BOD, blepharospasm-oromandibular dystonia; HAMA, the Hamilton Anxiety Rating Scale; HAMD, the Hamilton Depression Rating Scale; BoNT: botulinum toxin treatment.

### Quality of life assessment

**Figure 1:**
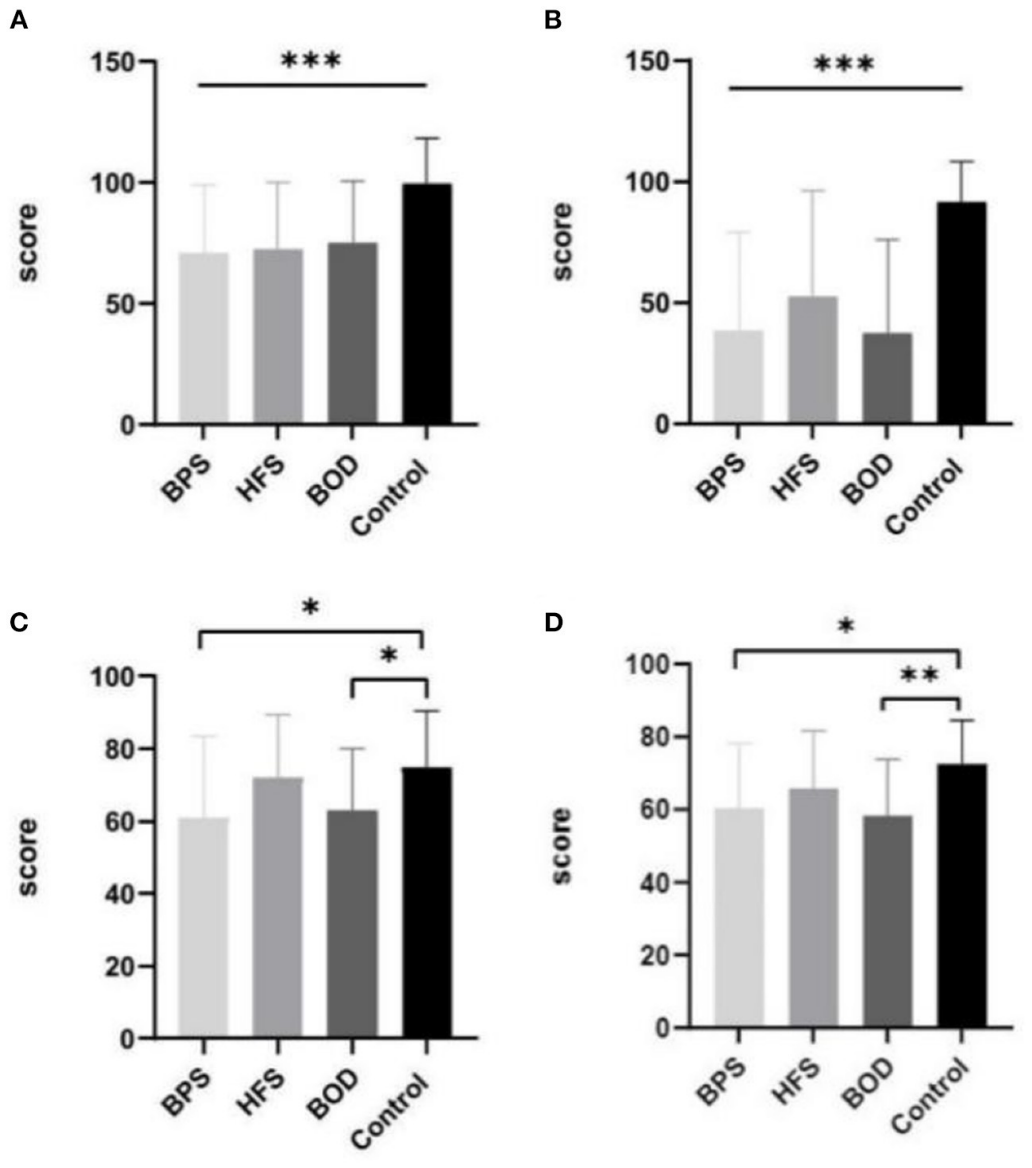
The comparison of mean SF-36 scores between groups. Compare to the control group, the mean scores in BPS, HFS, and BOD for SF **(A)** and RP **(B)** were significantly lower (*P* < 0.001). The VT score **(C)** was lower in the BPS group and BOD groups than in the control group (*P* < 0.05). The MH score **(D)** in the BPS (*P* < 0.05) and BOD (*P* < 0.01) groups significantly differed from the control group.

Patients with craniofacial movement disorders had lower mean SF-36 scores than controls in multiple dimensions. Regarding the SF-36 variables, RP, energy (i.e., vitality, VT), SF, RE, and MH scores were significantly lower in the movement disorders groups than in the control group (all *p* < 0.05). The mean scores for SF (*F* = 8.755, *p* = 0.0004) and RP (*F* = 14.460, *p* = 0.0004) significantly differed between those with and without movement disorders. VT scores were poorer in the BPS and BOD groups than in the control group (*F* = 13.833, *p* = 0.024, and *F* = 11.83, *p* = 0.036, respectively). MH scores in the BPS and BOD groups significantly differed from those in the control group (*F* = 12.133, *p* = 0.017, and *F* = 14.267, *p* = 0.003, respectively) (Figure 1).

### Depression and anxiety assessment

**Figure 2:**
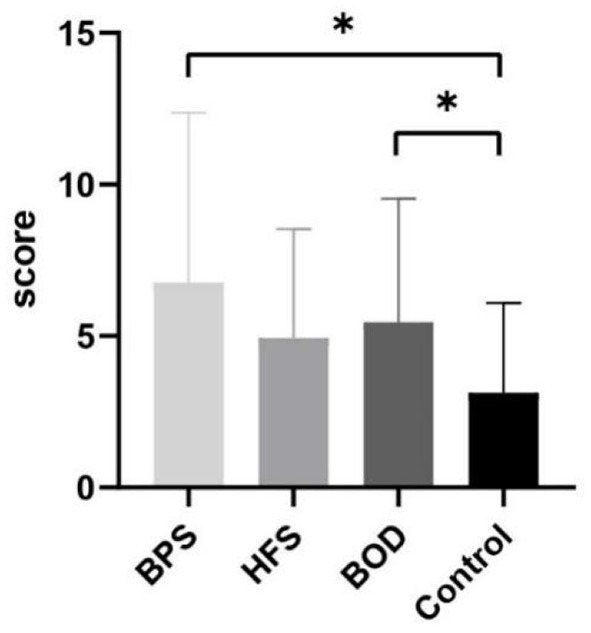
The comparison of mean HAMA scores between groups. Compare to the control group, BPS (*P* = 0.021) and BOD (*P* = 0.020) has significantly higher HAMA score. * provides significance *P* < 0.05.

Comorbid depression was present in 37 (41.11%) patients. Twenty-nine women (78.38%) and seven men (18.91%) were mildly depressed, whereas one woman (2.70%) had moderate depression. The most common manifestation of depression was sleep disorder; 33 patients (36.67%) reported insomnia, 23 (25.56 %) were light sleepers and had dreams more frequently, and 17 (18.89 %) woke up early. Thirty patients (33.33%) had anxiety, of whom 25 were women (83.33%) and 5 were men (16.67%). Among them, 23 patients (76.67%) had mild anxiety, 6 (20%) had moderate anxiety, and 1 (3.33%) had severe anxiety. The prevalence of depression did not differ between groups; however, the prevalence of anxiety significantly differed across the four groups (H=6.123, *p* = 0.009). The HAMA scores of the BPS (*p* = 0.021) and BOD (*p* = 0.020) groups significantly differed from those of the control group (Figure 2). Further, HAMD scores correlated with disease duration (β = −0.270, *p* = 0.010) in the multiple linear regression model as disease duration increased and depression scores decreased. Additionally, HAMA scores correlated with HAMD scores (β = 0.818, *p* < 0.001).

### Stigma assessment

Nineteen of 90 patients with craniofacial movement disorders (21.11%) reported feeling stigmatized (Table 2); 22 (24.44%) felt they did not receive more attention from family members, and 7 (7.78%) kept their conditions a secret. Regarding external stigma, one patient reported separating from their partner due to craniofacial movement disorders. Unemployment rate was 25.56% in these patients, and nine patients (10%) were terminated from their jobs.

**Table 2:** Stigma-related questions (*n* = 90).

	**Question**	**Percentage (%)**	
		**Yes**	**No**
1	Did you ever perceive stigmatization from people without craniofacial movement disorders?	21.11%	78.89%
2	Do you think that your family members are more protective of you?	75.56%	24.44%
3	Do your friends know about your craniofacial movement disorders?	92.22%	7.78%
4	Did your partner ever break up with you because you have craniofacial movement disorders?	1.11%	98.89%
5	Have you ever been fired from a job because you have craniofacial movement disorders?	10.00%	90.00%
6	Have you ever had difficulty finding a job because you have craniofacial movement disorders?	25.56%	74.44%
7	Do you think that people's attitudes changed when they learned that you have craniofacial movement disorders?	28.89%	71.11%

Employment-seeking activity significantly differed between those with and without movement disorders (*X*^2^ = 13.750, *p* = 0.003; Table 3). Patients with BPS and HFS reported more difficulty finding employment (Bonferroni correction, *X*^2^ = 7.124, *p* = 0.008) and were more likely to perceive attitude changes (*X*^2^ = 14.06, *p* = 0.002). Twenty-six patients (28.89%) thought that people's attitudes worsened when they learned about the patient's disease. We did not find correlations between stigma and sex, age, or disease duration.

**Table 3:** Stigma comparisons [*n* (%)].

Question^*^	**1**	**2**	**3**	**4**	**5**	**6**	**7**
	Yes *n* (%)	Yes *n* (%)	Yes *n* (%)	Yes *n* (%)	Yes *n* (%)	Yes *n* (%)	Yes *n* (%)
BPS	5 (16.67)	25 (83.33)	28 (93.33)	0 (0.00)	4 (13.33)	10 (33.33)	12 (40.00)
HFS	7 (23.33)	21 (70.00)	29 (96.67)	1 (3.33)	4 (13.33)	10 (33.33)	8 (26.67)
BOD	7 (23.33)	22 (73.33)	26 (86.67)	0 (0.00)	1 (3.33)	3 (10.00)	6 (20.00)
Control	4 (13.33)	24 (80.00)	21 (70.00)	0 (0.00)	0 (0.00)	1 (3.33)	0 (0.00)
χ^2^	1.452	0.982	10.962	3.025	5.954	13.750	14.730
*P*	0.693	0.806	0.012	0.388	0.100	0.003	0.002

BPS, blepharospasm; HFS, hemifacial spasm; BOD, blepharospasm-oromandibular dystonia.

* See Table 2.

### Factors influencing the SF-36 score of patients with craniofacial movement disorders

Using correlation analysis, the eight dimensions of SF-36 were separately correlated with education level, disease duration, HAMD score, and HAMA score, all with statistically significant differences (*p* < 0.05) (Table 4). Stepwise linear multiple regression analysis showed that HAMD scores correlated with GH, VT, SF, RE, and MH after adjusting for education level, disease duration, and HAMA score (all *p* < 0.05). The HAMA score correlated with BP after adjusting for education level, disease duration, and HAMD score (β = −0.038, *p* = 0.032) (Table 5).

**Table 4:** Factors correlation with the eight domains of SF-36.

	**Sex**		**Age**		**Education**		**Disease duration**		**HAMD**		**HAMA**	
	**β**	***p***	**β**	***p***	**β**	***p***	**β**	***p***	**β**	***p***	**β**	***p***
GH	0.075	0.484	−0.098	0.357	0.282	0.007	0.295	0.005	−0.598	0.000	−0.549	0.000
VT	0.159	0.135	0.005	0.964	0.179	0.091	0.295	0.005	−0.586	0.000	−0.472	0.000
SF	−0.018	0.870	−0.086	0.422	0.219	0.038	0.159	0.133	−0.420	0.000	−0.288	0.006
PF	−0.128	0.231	−0.150	0.158	0.080	0.453	0.284	0.007	−0.345	0.001	−0.274	0.009
RP	−0.008	0.940	−0.009	0.929	0.187	0.078	0.325	0.002	−0.369	0.000	−0.267	0.011
BP	0.078	0.464	−0.195	0.066	0.158	0.137	0.200	0.059	−0.281	0.007	−0.334	0.001
RE	−0.102	0.339	0.011	0.917	0.282	0.007	0.213	0.044	−0.405	0.000	−0.255	0.015
MH	0.193	0.068	0.074	0.488	0.200	0.059	0.306	0.003	−0.484	0.000	−0.384	0.000

GH, General Health; VT, Vitality; SF, Social Function; PF, Physical Function; RP, Role-Physical; BP, Bodily Pain; RE, Role-Emotional; MH, Mental Health; HAMA, the Hamilton Anxiety Rating Scale; HAMD, the Hamilton Depression Rating Scale.

**Table 5:** Multiple linear regression analysis of depression, anxiety, and SF-36.

	**GH**		**VI**		**SF**		**PF**		**RP**		**BP**		**RE**		**MH**	
	**β**	***p***	**β**	***p***	**β**	***p***	**β**	***p***	**β**	***p***	**β**	***p***	**β**	***p***	**β**	***p***
**HAMD**
Model 1	−0.598	0.000	−0.586	0.000	−0.420	0.000	−0.345	0.001	−0.369	0.000	−0.281	0.007	−0.405	0.000	−0.484	0.000
Model 2	−0.040	0.047	−0.055	0.002	−0.029	0.007	−0.016	0.162	−0.012	0.096	0.010	0.580	−0.021	0.008	−0.042	0.026
**HAMA**
Model 1	−0.549	0.000	−0.472	0.000	−0.288	0.006	−0.274	0.009	−0.267	0.011	−0.334	0.001	−0.255	0.015	−0.384	0.000
Model 2^*^	−0.036	0.076	−0.004	0.838	0.009	0.437	−0.002	0.845	0.001	0.911	−0.038	0.032	0.008	0.342	−0.011	0.590

Model 1 is a univariate regression model; model 2 was adjusted for education, disease duration and HAMA score; model 2^*^ was adjusted for education, disease duration, and HAMD score.

GH, General Health; VT, Vitality; SF, Social Function; PF, Physical Function; RP, Role-Physical; BP, Body Pain; RE, Role-Emotional; MH, Mental Health; HAMA, the Hamilton Anxiety Rating Scale; HAMD, the Hamilton Depression Rating.

## Discussion

This study focused on the mental health and disease-related QoL of Chinese patients with craniofacial movement disorders. Patients with craniofacial movement disorders have a high rate of depression and anxiety, as well as considerable stigmatization, which lower the patient's QoL.

Regarding mental health assessment, our findings correspond to those of Fabbrinid et al. (4), who reported that patients with BPS and BOD are more susceptible to emotional disorders compared with HFS. In our study, BPS and BOD groups had higher HAMA and HAMD scores. Women had higher scores for anxiety and depression, which could be explained by appearance-related anxiety (31). We found no differences in the prevalence of depression among the three movement disorders groups. The most common problem regarding depression was poor sleep quality. A previous study indicated that patients with BPS may experience insomnia-related complaints (32). Dystonic movement and associated pain may increase latency in sleep onset and the number of awakenings, reducing total sleep time and sleep efficiency. HAMD and HAMA scores were correlated, suggesting that depression and anxiety are often comorbid. The multiple linear regression analysis indicated that patients had higher depression scores in the early disease stages. Therefore, more attention should be paid to the mental health of patients who visit the clinic for the first time. Psychotherapeutic approaches, such as cognitive behavioral therapy (CBT), can help relieve anxiety and muscle tension, leading to the potential for improved outcomes of motor symptoms (33).

In addition to depression and anxiety, in our study, some patients showed signs of stigma. Research on health-related stigma is lacking worldwide, especially in developing countries (34). Stigma, on the other hand, is not rare in chronic diseases. Patients with stroke (35), epilepsy (36), and Parkinson's disease (37) reported experiencing some form of stigma. Rinnerthaler et al. (38) also reported that patients with cranial and cervical movement disorders were less self-confident and subject to serious prejudice and stigmatization. For BPS and HFS, appearance concerns and beliefs about the consequences of illness are important predictors of stigma (39). There are two primary classifications of stigma: internalized or “felt” stigma (associated with low self-esteem or self-doubt) and enacted stigma (experiencing negative public attitudes) (40). In this study, we found that stigma associated with craniofacial movement disorders mainly manifested as enacted stigma (i.e., by the public). The effect of social stigma among unemployed individuals has been underestimated (41). In comparison to the general population, patients with craniofacial movement disorders found it more difficult to achieve basic social rights such as finding employment. Some patients experienced bias and were rejected for employment. Employers often hold negative attitudes toward people with poor appearances, and self-stigma and the “why try” effect (42) after rejection can lead to low motivation to find employment. Patients with BPS and HFS were more susceptible to the negative attitudes of people around them concerning their condition. Collectively, these issues indicate that stigma occurs at multiple levels, ranging from intrapersonal to interpersonal, and to various structural levels (43). Most patients with craniofacial movement disorders do not feel different from unaffected people and will not deliberately conceal the disease from their friends. Developing self-adaptation and self-acceptance could account for this. In clinical diagnosis and therapy, stigma is largely neglected, resulting in poor treatment outcomes (44).

QoL has been considered when evaluating chronic disease therapy (45). All movement disorders groups had lower RP and SF scores. Mental health has an impact on each aspect of QoL. Negative emotional states, such as depression, anxiety, and perceived stigma, are the factors associated with quality of life (39). We found that depression had a greater impact on QoL than anxiety. In addition to the effects of education level, depression played a role in decreased social function, which mainly manifested in employment seeking, as shown in the stigma questionnaire. Studies have shown that patients with mental illnesses such as depression are less motivated to seek employment (46). Stigma also limits social opportunities and resources (30, 32, 47). As a result of involuntary facial movement and enacted stigma, most patients face limitations in job selection, and extra effort is required to complete the same work. Majority of the patients reduced their time for social activities, restricting their activities. In addition, disease duration correlated with GH, VT, PF, RP, and MH scores. Patients first presenting with the disease or with a short course of illness experienced helplessness on encountering the disease. Together, these findings suggest that movement disorder-associated mental health may differentially affect QoL indicators.

Our study has some limitations. First, this was a cross-sectional study that failed to compare changes in stigma after treatment with botulinum toxin. Second, since there is no standard measurement for stigma in craniofacial movement disorders, we selected a questionnaire based on epilepsy research. Many studies have shown that the majority of people with epilepsy experience stigma (48). Although the questionnaire has good internal consistency, this approach may not adequately demonstrate the severity of stigma in craniofacial movement disorders patients. Also, patients may not be honest with strangers about their experiences with stigma during face-to-face questionnaires. Due to different physiopathology, some researchers have proposed whether HFS belongs to facial dystonia, or as a type of facial disfigurement. Considering that we performed a preliminary evaluation of anxiety and depression, we did not choose the Structured Clinical Interview for DSM-IV Dissociative disorders (SCID-D) to further assess personality disorders. The sleep items of HAMD are not sufficient to describe the patient's sleep problems, and we will use specialized sleep scales for the follow-ups.

In conclusion, in addition to the disease itself, some patients with craniofacial movement disorders experience psychiatric problems, which can lead to a multidimensional decline in QoL, affecting both physical and social functions. Therefore, understanding the psychological aspects of movement disorders and incorporating psychiatric evaluations into future clinical and research assessments of craniofacial movement disorder patients may help us better understand how the brain works and develop more effective treatments.

## Data availability statement

The original contributions presented in the study are included in the article/Supplementary material, further inquiries can be directed to the corresponding author.

## Ethics statement

Written informed consent was obtained from the individual(s) for the publication of any potentially identifiable images or data included in this article.

## Author contributions

MY, CD, and WZ conceived of the presented idea. GL and JL (2nd author) verified the analytical methods. ZO, YL, and YG contributed to sample preparation. MY and JL (6th author) wrote the original draft. CD, GL, and YC reviewed and edited the manuscript. WZ, YW, and JZ helped supervise the project. All authors provided critical feedback and helped shape the research, analysis, and manuscript.

## Funding

This study was supported by grants from the Natural Science Foundation of Guangdong Province, China (No.2021A1515010600); The National Natural Science Foundation of China (No. 81500994); The Natural Science Foundation of China (No. 82101399); The Natural Science Foundation of China (81901077); The Basic and Applied Basic Research Foundation Natural Science Foundation of Guangdong Province (2021A1515012216); The Kelin Star Talent Support Program of the First Affiliated Hospital, Sun Yat-sen University (R08014); Medical Scientific Research Foundation of Guangdong Province, China (A2022221); The Guangdong Provincial Key Laboratory of Diagnosis and Treatment of Major Neurological Diseases (2020B1212060017), Guangdong Provincial Clinical Research Center for Neurological Diseases (2020B1111170002), Southern China International Joint Research Center for Early Intervention and Functional Rehabilitation of Neurological Diseases (2015B050501003 and 2020A0505020004), Guangdong Provincial Engineering Center for Major Neurological Disease Treatment, Guangdong Provincial Translational Medicine Innovation Platform for Diagnosis and Treatment of Major Neurological Disease, Guangzhou Clinical Research and Translational Center for Major Neurological Diseases (201604020010).

## Conflict of interest

The authors declare that the research was conducted in the absence of any commercial or financial relationships that could be construed as a potential conflict of interest.

## Publisher's note

All claims expressed in this article are solely those of the authors and do not necessarily represent those of their affiliated organizations, or those of the publisher, the editors and the reviewers. Any product that may be evaluated in this article, or claim that may be made by its manufacturer, is not guaranteed or endorsed by the publisher.

## Abbreviations

QoL: quality of life
HFS: hemifacial spasm
BPS: blepharospasm
BOD: blepharospasm-oromandibular dystonia
HAMA: Hamilton Rating Scale for Anxiety
HAMD: Hamilton Rating Scale for Depression
RP: Role-Physical
VT: Vitality
SF: Social Function
RE: Role-Emotional
MH: Mental Health
GH: General Health
BP: Body Pain.
